# Use of Postmortem Human Dura Mater and Scalp for Deriving Human Fibroblast Cultures

**DOI:** 10.1371/journal.pone.0045282

**Published:** 2012-09-27

**Authors:** Lindsay A. Bliss, Malik R. Sams, Amy Deep-Soboslay, Renee Ren-Patterson, Andrew E. Jaffe, Josh G. Chenoweth, Amritha Jaishankar, Joel E. Kleinman, Thomas M. Hyde

**Affiliations:** 1 Section on Neuropathology, Clinical Brain Disorders Branch, Division of Intramural Research Programs, National Institute of Mental Health, National Institutes of Health, Bethesda, Maryland, United States of America; 2 Lieber Institute for Brain Development, Johns Hopkins Medical Campus, Baltimore, Maryland, United States of America; University of South Florida, United States of America

## Abstract

Fibroblasts can be collected from deceased individuals, grown in culture, reprogrammed into induced pluripotent stem cells (iPSCs), and then differentiated into a multitude of cell types, including neurons. Past studies have generated iPSCs from somatic cell biopsies from either animal or human subjects. Previously, fibroblasts have only been successfully cultured from postmortem human skin in two studies. Here we present data on fibroblast cell cultures generated from 146 scalp and/or 53 dura mater samples from 146 postmortem human brain donors. In our overall sample, the odds of successful dural culture was almost two-fold compared with scalp (OR = 1.95, 95% CI: [1.01, 3.9], p = 0.047). Using a paired design within subjects for whom both tissues were available for culture (n = 53), the odds of success for culture in dura was 16-fold as compared to scalp (OR = 16.0, 95% CI: [2.1–120.6], p = 0.0007). Unattended death, tissue donation source, longer postmortem interval (PMI), and higher body mass index (BMI) were associated with unsuccessful culture in scalp (all p<0.05), but not in dura. While scalp cells proliferated more and grew more rapidly than dura cells [F (1, 46) = 12.94, p<0.008], both tissues could be generated and maintained as fibroblast cell lines. Using a random sample of four cases, we found that both postmortem scalp and dura could be successfully reprogrammed into iPSC lines. Our study demonstrates that postmortem dura mater, and to a lesser extent, scalp, are viable sources of living fibroblasts for culture that can be used to generate iPSCs. These tissues may be accessible through existing brain tissue collections, which is critical for studying disorders such as neuropsychiatric diseases.

## Introduction

Living fibroblasts can be cultured from human cadavers for use in induced pluripotent stem cell (iPSC) generation. This is particularly valuable to test the biological fidelity of the mature cells derived from ordinarily inaccessible tissue and then compared back to the primary cells from the same individual. For example, a number of studies have generated cells with a neuronal phenotype from fibroblasts derived from living subjects, as previously reviewed [1]. Other than olfactory epithelium, it is virtually impossible to biopsy neurons to compare to those derived from fibroblasts from the same individual [2].

Fibroblasts can be converted into iPSCs, which can further be differentiated into neurons [3], [4]. Two groups to date have cultured fibroblasts from postmortem human dermal tissue samples [5], [6]. Meske and colleagues generated human fibroblast cultures from postmortem abdominal skin biopsies [5]. In addition, Hjelm and colleagues generated iPSCs from fibroblasts using skin samples from a single autopsy case of a 75 year-old whole body donor [6]. Additionally, fibroblasts were recently cultured from postmortem ear tissue of sheep [7]. Culturing of postmortem tissue samples offers another avenue of study for human disease. Here we highlight a novel and potentially important resource not only for those involved in tissue collection, but also for the field of cellular reprogramming. The validity of studies of fully differentiated neurons derived from iPSCs is based on the premise that the derived differentiated neurons exhibit most if not all of the biological characteristics of their natural counterparts.

We first set out to determine whether it was possible to generate fibroblasts from scalp and/or dura mater samples from a large consecutive series of postmortem human brain donors, as a way to provide a source of cells for future studies of neuronal phenotypes in schizophrenia and related disorders. We then determined which tissue was most optimal for tissue culturing. Next, we examined whether certain demographic or tissue characteristics were associated with culturing of fibroblasts from postmortem human scalp or dura, in an effort to determine which tissue might be used in the future by our laboratory and others. Moreover, we used postmortem scalp and dura fibroblasts to generate iPSCs for several cases.

## Results

### Fibroblast Characterization and Proliferation

**Figure 1:**
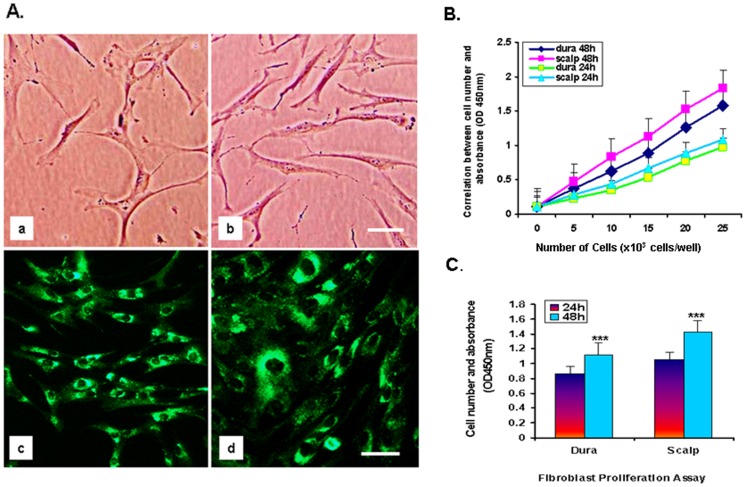
Fibroblast characterization: FSP-1 protein expression by immunofluorescence staining and cell proliferation assay in dura and scalp. A. The morphology of postmortem fibroblast cells generated from (a) dura and (b) scalp. Cultured cells from both sources macroscopically looked similar to what is seen in living skin fibroblast cells, with more enriched cytoplasm and spindle-shaped nuclei under phase-contrast microscopy. Cells from (c) dura and (d) scalp express cytoplasmic Fibroblast Specific Protein-1 (FSP-1) (green). Original scale bars = 35 µm. **B.** Results from cell proliferation assay in 8 fibroblast cell lines (dura and scalp from 4 individuals) in five different densities. Cell viability was determined in 24 hrs and 48 hrs by WST-8 assay. Values are the mean of results from six wells. Bars ± SE. Scalp fibroblast cell lines grew 1.27-fold faster in the same period than dura fibroblast cells. **C.** Differences in cell proliferation between scalp and dura by one-way ANOVA; scalp cell growth was significantly more rapid than dura cell growth at 24 hr and 48 hr intervals [F (1, 46) = 12.94, p<0.008].

When fibroblast cells were first generated from postmortem scalp and dura, we found that they initially began to differentiate more slowly (around 10 days) than our lab’s previous experience with live skin-generated fibroblast cells, which differentiate in 5–7 days. The morphology of scalp and dura postmortem cells looked similar to what has been previously seen in living skin fibroblast cells. The postmortem scalp cells showed more elongation than dura cells, but after 3 weeks both scalp and dura showed spindle-shaped nuclei in higher magnification under phase-contrast microscopy (Figure 1-A, a–b). Secondly, we used immunofluorescence staining to determine the presence of fibroblast surface protein 1 (FSP-1) (Figure 1-A, c–d) and human thymic fibroblast protein (TE7) (Figure S1) in cultured dura and scalp cells. We found significant expression of both FSP-1 and TE7 proteins in the cytoplasm of fibroblast cells. The staining results revealed that the postmortem scalp and dura fibroblast cells exhibit similar protein expression as previously observed in live skin fibroblasts. Finally, we further evaluated whether there was a difference in cell proliferation between postmortem scalp and dura fibroblast cultures. We tested this using a CCK-8 proliferation assay kit on 8 fibroblast cell lines (scalp and dura from 4 individuals), each assessed at 24 and 48 hrs. The results showed that all 4 scalp fibroblast cell lines grew 1.27-fold more cells in the same time period than dura fibroblasts (Figure 1-B). One-way ANOVA showed that scalp cell lines proliferated significantly more than dura cell lines in the same time interval [F (1, 46) = 12.94, p<0.0008) (Figure 1-C).

### Scalp vs. Dura

**Figure 2:**
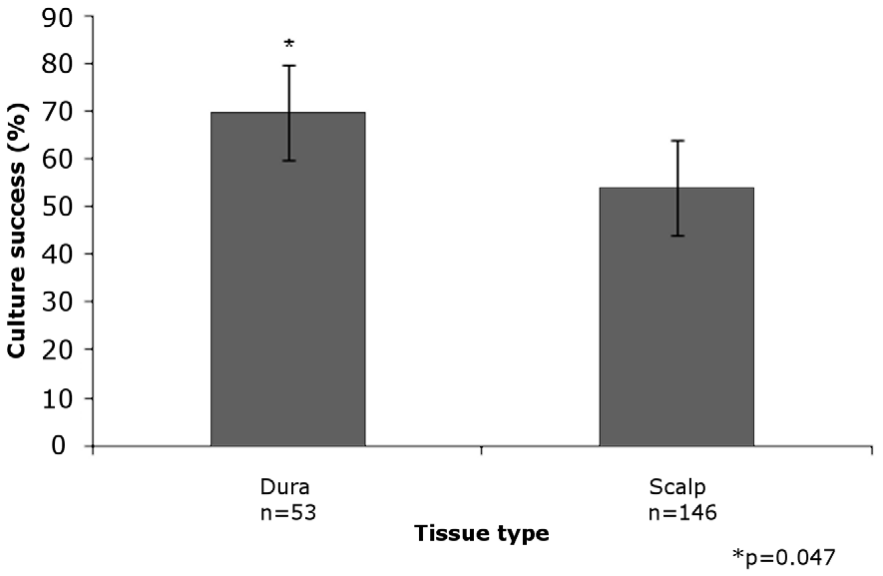
Percentage of successful growth in dura (n = 53) vs. scalp (n = 146) samples. Sample of 146 scalp and 53 dural specimens. The odds of successful culture in dura were nearly 2-fold compared to scalp.

146 scalp and 53 dural samples from postmortem brain donors were analyzed. The odds of successful culture in dura was almost 2-fold compared to scalp (OR = 1.95, 95% CI: [1.01, 3.9], p = 0.047) (Table 1; Figure 2). All 53 individuals with dural samples also had scalp samples. First, we compared individuals where culture failed in both tissue types (N = 15) to those who had successful culture in both (N = 21). None of our measured covariates could explain the difference in overall culture success. Then, we added individuals with successful dural culturing but with unsuccessful scalp culturing (N = 16), and treated these individuals as an intermediary group in subsequent additive models. Again, none of these covariates could explain the difference we observed in culture success differences by tissue type.

**Table 1:** Odds ratio of culture success: dura vs. scalp matched pairs (n = 53).

		Dura	
		No	Yes
Scalp	No	15	16
	Yes	1	21

No = not successful, Yes = successful.

The most informative tissue pairs came from those individuals that were discordant for culture success (i.e., successful culturing in one tissue but not the other). Given the paired design, we employed McNemar’s Test for Marginal Heterogeneity, which suggested a strong effect of dural culturing compared to scalp (p = 0.0007). The odds of successful culture was 16-fold for dura compared to scalp (Matched odds ratio: 16.0, 95% CI: 2.1–120.6).

Cell culturing was successful in PMIs as high as 66.5 hrs in dura, and 73.5 hrs in scalp; however, when outliers were removed, culturing was successful in PMIs up to 58 hrs in dura and 55 hrs in scalp (Figure S2).

### Scalp Only

We then stratified the data to look for tissue-specific effects (Table 2). Attended death was independently associated with the odds of culture success in scalp (OR = 0.4 [0.199–0.783], p = 0.008), and increased after adjusting for tissue source (OR = 3.16 [1.54–6.69], p = 0.002). The tissue donation source of the scalp sample was significantly associated with culture success, with the odds of success 2.37 fold (95% CI =  [1.2–4.76]) greater at the VA rather than the DC medical examiner. In a multivariate logistic regression model with postmortem interval (PMI), adjusting for tissue donation source, both PMI and tissue donation source were independently associated with culture success at similar levels as the univariate associations. When only samples with attended deaths were retained in the analysis (N = 89), the effect sizes were approximately the same as when all samples were included. Tissue donation source was still independently associated with culture success in attended death samples (OR = 2.4 [1.2–4.76], p = 0.014). In addition, PMI was negatively associated with culture success in scalp (OR = 0.97 [0.946–0.994], p = 0.019). Body mass index (BMI) was negatively associated with culture success in adult scalp samples (OR = 0.96 [0.928–0.998], p = 0.047), but the negative effect was attenuated when extremely obese (BMI >60, n = 4) individuals influencing the regression were excluded (OR = 0.97 [0.92–1.01], p = 0.122) (Figure S2). In the full set of scalp samples, race was not associated with culture success in scalp (OR = 1.42 [0.66–3.08], p = 0.33). Scalp fibroblast cultures were more prone to fungal and bacterial contamination using the India ink test. This was identified by observation of an increase in turbidity, a change in color of the culture media, and by microscopic inspection [8].

**Table 2:** Demographic and sample parameters of successful culture in scalp (n = 146).

	OR	95% CI	*p*-value
**Age (yrs)**	0.99	0.967–1.01	0.270
**Brain pH**	3.2	0.634–16.9	0.160
**Race (AA vs. W)**	1.42	0.66–3.08	0.330
**PMI (hrs)**	0.97	0.95–0.99	0.019*
**BMI**	0.96	0.93–0.998	0.047*
**Sex (F vs. M)**	1.6	0.807–3.07	0.190
**Smoker (No vs. Yes)**	0.86	0.448–1.66	0.660
**Substance-Related** **Death (No vs. Yes)**	0.83	0.415–1.68	0.610
**Cardiac-Related** **Death (No vs. Yes)**	1.3	0.648–2.44	0.500
**Tissue Source** **(VA vs. DC)**	2.4	1.2–4.76	0.014*
**Attended Deaths** **(Attended vs.** **Unattended)**	2.51	1.28–5.01	0.008*
**Positive Ethanol Tox** **(Negative vs. positive)**	0.83	0.353–1.96	0.670

OR = odds ratio, AA = African-American, W = White, PMI = postmortem interval, BMI = body mass index, F = female, M = male, VA = Virginia, DC = District of Columbia; Tox = toxicology testing in blood or vitreous humor; * = p<0.05.

### Dura Only

In dura, none of the measured covariates were significantly associated with culture success. There was no effect of attended deaths, tissue donation source, PMI, or BMI on dural culture after performing the same analyses as done in scalp.

### Reprogramming

**Figure 3:**
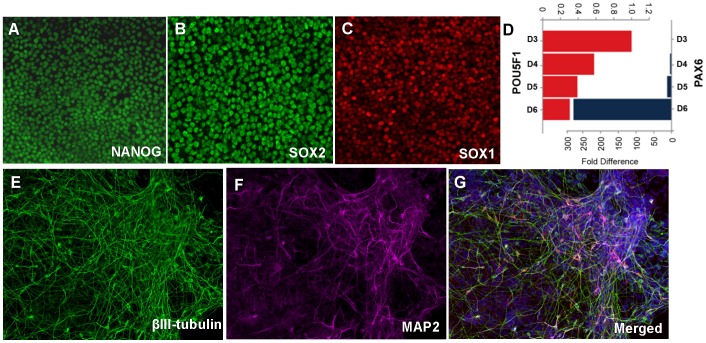
iPSCs generated from one dura fibroblast line express pluripotency markers and differentiate to neuronal fates. Undifferentiated iPSCs express pluripotency markers NANOG (A) and SOX2 (B). Upon neural differentiation, these cells express neuroectoderm marker SOX1 (C). Temporal gene expression analysis also shows a decrease in pluripotency marker OCT4 (POU5F1) and an increase in *PAX6* expression by quantitative RT-PCR (D). iPSC-derived neurons express βIII-tubulin and MAP2 (E–G).

To test the potential of both scalp and dura derived fibroblasts as sources of iPSCs, we used a polycistronic lentiviral approach to express OCT4, KLF4, C-MYC and SOX2 as described in Sommer and Mostoslavsky [9]. We infected scalp and/or dura derived fibroblasts from four randomly chosen postmortem subjects. Both scalp and dura sources yielded colonies that were picked and expanded based on morphology consistent with human pluripotent stem cells. Fourteen independent iPSC lines in total were expanded and cryopreserved. These iPSC lines express pluripotency markers including OCT4 (POU5F1), NANOG and SOX2 by immunocytochemistry and mRNA expression (Figure 3). To test the differentiation potential of these lines to neuronal fates, we used the protocol described in Chambers and Studer [10]. Upon neuronal induction, the iPSCs showed a decrease in expression of pluripotency-associated genes, accompanied by an increase in lineage-specific genes such as SOX1 and PAX6. These neuroectodermal cells could be further differentiated into mature neurons as evidenced by the expression of βIII-tubulin and MAP2.

## Discussion

This study presents the use of two novel tissues, human scalp and dura mater, as successful sources of cultured fibroblasts. It is the first study to compare potential sources of fibroblasts from postmortem human samples, and unlike previous reports, provides data from a large cohort of well-characterized subjects. As a result, this enabled us to examine the relationships between demographic and perimortem factors with respect to culture success, as well as to observe any possible differences in fibroblast growth or iPSC generation between the two postmortem tissues.

The odds of culture success were 16-fold greater in dura as compared with scalp in within-subjects scalp-dura pairs. In scalp, PMI was inversely associated with successful cell growth; the lower the PMI, the more likely to be successfully cultured. Not surprisingly, as in organ transplantation, the sooner the tissue is harvested, the greater the viability. In scalp, having an attended death was also significantly associated with successful culturing, consistent with these deaths usually being associated with a shorter PMI. This finding agrees with previous studies that reported less successful culturing in individuals with a longer PMI [11]. We also found for scalp that successful culturing was associated with lower BMI; however, this finding appeared to be largely driven by a few outlying morbidly obese individuals (BMI>60) in our sample. This result may be related to metabolic abnormalities associated with obesity that have an impact on postmortem fibroblast viability.

Thus, we found that dural fibroblasts were most readily cultured. Perimortem and postmortem factors appeared to play no discernible role in predicting the success of these dural cultures. Scalp cultures also had more contaminations than dural cultures. Dural fibroblasts naturally grow in a relatively sterile environment whereas scalp fibroblasts grow in an environment rich in microbes. Moreover, the scalp is open to other potentially detrimental environmental factors that may inhibit growth in culture such as ultraviolet radiation, while dura mater is from a protected environment within the skull. It is worth noting that scalp and dural tissue required different collection and handling procedures in the first few days of tissue culturing; therefore, it is possible that the different culturing techniques required for each tissue may have adversely influenced scalp cell culture growth.

Interestingly, even though dura fibroblasts were significantly more successful in culture overall, scalp cell lines proliferated significantly more rapidly than dura cell lines. This finding suggests that even though scalp and dura yield fibroblast cultures, there may be epigenetic modifications giving scalp fibroblasts greater proliferative potential. It is well known that the scalp is outside of the skull and comprises hair-bearing skin, lying on dense, highly vascular connective tissue with denser fibroblast cells. Furthermore, we have noted the large vessels seen when scalp tissue was processed for cultures in this study. However, the dura mater is a much different tissue, which is a layer on an inner membrane of connective tissue and contains more collagen. Therefore, the more rapid proliferation of scalp fibroblasts might have been predicted, as the skin cells turn over at a much faster rate than the dura.

Most importantly, we demonstrated that fibroblasts from both postmortem human scalp and dura can be differentiated into iPSCs. Admittedly, a larger number of reprogramming experiments will need to be performed to determine if there is a bias between scalp and dura with respect to the reprogramming success. For example, Wakao and colleagues recently reported that Muse (i.e., multilineage-differentiating stress-enduring) cells were a more optimal source for iPSCs as opposed to non-Muse cells in dermal fibroblast cultures [12]. Whether or not Muse cells are present in our cultures has yet to be determined.

In conclusion, this study shows that two postmortem human tissues, dura and scalp, can serve as sources for fibroblast cultures, and subsequent iPSC generation. Increased culture success in scalp is seen in samples from individuals with an attended death, tissue collected from the VA medical examiner source, shorter PMIs, and lower BMIs. While our findings particularly support the use of dural samples for postmortem fibroblast cultures due to their covariate independence, both tissues were viable sources for fibroblasts and iPSCs. As patient-specific iPSCs are developed to study psychiatric and neurological disorders, it will be critical to define the equivalence of iPSC neuronal derivatives to primary human neurons. Our results suggest that iPSCs derived from postmortem fibroblasts with matched donor brain tissue could be a potentially exciting avenue for further exploring neuropsychiatric disorders.

## Materials and Methods

### Tissue Collection

Postmortem human brain samples (n = 146) were collected through consecutive donations at two local area medical examiners (Commonwealth of Virginia, Northern District and Washington, DC offices) at the Clinical Brain Disorders Branch (CBDB), Division of Intramural Research Programs (DIRP), NIMH. Informed consent was obtained verbally from the legal next-of-kin for every case using a telephone script, and was both witnessed and audiotaped, as outlined in the IRB approved NIMH protocol 90-M-0142. Cases were characterized as previously described [13]. Deaths were coded as either attended (e.g., witnessed collapse or coding in hospital setting) or unattended. A telephone screening was conducted on the day of donation to gather basic demographic, medical, and clinical histories on every donor to determine a preliminary psychiatric or neurological diagnosis. Postmortem interval (PMI) was calculated as time elapsed between death and tissue freezing in hours. Body mass index (BMI) was calculated from height and weight at autopsy, and obesity was defined by a BMI>30. Microscopic and macroscopic neuropathological examinations were performed on every case to screen for neurological pathology. Toxicological analysis in blood was performed for every case by the medical examiner’s office to screen for illicit, and in some cases, basic drugs.

Dural and scalp tissue were collected at autopsy. We received the tissues from the autopsy room in separate bags; one containing cerebral dura and the other a 2 in by 4 in scalp segment with attached hair. The dura was checked for pathologic changes such as tumor or hemorrhage. Both the dura mater and scalp were cut into 0.2 in by 0.5 in pieces, transported in vials containing 2 ml of antibiotic transfer media, then returned to the lab, and the culture procedure was immediately started. For detailed demographic data on scalp and dura tissue donors, please see Table 3.

**Table 3:** Sample Demographics.

	Age (yrs)	Brain pH	PMI (hrs)	Sex (%M)	Race (%W/%AA)
Scalp Samples (n = 146)	45.3±15.7	6.46±0.21	31.7±14.1	59.60%	68.5%/24.7%
Dural Samples (n = 53)	43.6±18.4	6.48±0.19	32.2±12.3	60.40%	67.9%/24.5%

PMI = postmortem interval, M = male, W = White, AA = African-American.

### Scalp and Dura Cultures

The scalp sample was washed with a phosphate buffered saline solution (pH 7.2) three times, and fat and hair tissues were cut away. The dissected scalp sample was placed epidermis side down and floated with Dispase II enzyme solution (2.4 units/ml in HBSS, Dispase II enzyme, Cat#04942078001, Roche), which is a proteolytic enzyme used to separate the dermis from the epidermis by cleaving the basement membrane zone. It was then pipetted into a separate dish, and then covered with parafilm and foil overnight in a 4°C refrigerator. After 24 hrs, the scalp epidermis was peeled away from the dermis. The dermis was washed in a phosphate buffered saline, dried, and then cut into several 2–3 mm^3^ pieces that were placed in a special Easy Grip cell culture 35×10 mm dish (Cat#353801, BD). One drop of culture medium DMEM + Glutamax (Cat#10566, GIBCO) with 10% fetal bovine serum, 2% L-glutamine, 1% Penicillin/Streptomycin solution, as well as 1% Amphotericin and Gentamicin solution was added to each piece of scalp and placed into a 35 mm dish in the incubator (37°C and 5% CO^2^) for culturing.

Dural samples were washed like scalp samples. After washing, several 2–3 mm^3^ pieces were cut from dura and placed together in a culture dish. One drop of antibiotic media was added to each piece, then the culture dish was placed in the incubator for starting cultures. Each of the cultures was changed with fresh medium two to three times per week to promote fibroblast cell growth. The fibroblast cells started to grow around 7–14 days on average; however some individual samples took longer to grow, up to three weeks.

### Fibroblast Cell Cultures

When fibroblast cell growth reached ∼85–90% confluence under phase-contrast microscopic observation, these fibroblasts were trypsinized with 1 ml of a 0.25% trypsin solution (Cat#T4049, Sigma) and were incubated for 5 to 8 min, then given a 1∶1 ratio of media to trypsin. They were collected into 15 ml centrifuge tubes, and spun for 4 min at 1200 rpm. The supernatant was discarded, followed by the resuspension of the cells. These cells were transferred into 25-cm^2^ culture flasks and were kept in cultures for a 48 hr period, then transferred into 75-cm^2^ flasks for continued growth and reproduction. Finally, when the cells reached confluence 5 x10^6^ to 1×10^7^, they were trypsinized and spun, then transferred into freezing tubes for storage as a successful cell line and placed in the −80°C freezer, and later frozen in liquid nitrogen. Fibroblast cells were generated from individual scalp and dura for storage in 10 tubes, which were free from microbial contamination as determined by microscopic inspection.

### Cell Proliferation Assay

The fibroblast cells came from scalp and dura in five different densities (5, 10, 15, 20, 25 x10^3^ cells/well) and were seeded into normal growth culture medium into 96-well plates. Each of the 5 densities of fibroblast cultures was contained in 6 wells. Cell counts were determined after 24 hrs and 48 hrs by 2-(2-methoxy-4-nitrophenyl)-3-(4-nitrophenyl)-5-(2,4-disulphonyl)-2H-tetrazolium (WST-8) assay using a Cell Counting Kit (Cat# CK04-13, DoJin East, Tokyo, Japan) as described previously [14]. In brief, 10 µl of 3-(4,5-dimethylthiazol-2-yl)-2,5-diphenyltetrazolium bromide was added to each well. After incubation for another 2 hrs at 37°C, we determined cell proliferation colorimetrically by the optical density (OD) at 450 nm with a microplate reader (Beckman Coulter, DTX 880 Multimode detector, U.S.A). Each OD 450 was calculated according to the following formula: OD 450 = experimental well-blank well.

### Immunofluorescence Staining

Immunofluorescence staining was performed as previously detailed [15]. The fibroblast cells from each individual’s dura and scalp were cultured in 12-well plates for 48 hrs. The cells were washed with phosphate-buffer saline (PBS, pH 7.4) twice and fixed in cold methanol for 20 min at room temperature. Cells were washed with PBS three times, then the fixed cultures were incubated with blocking solution 5% normal goat serum (Cat# sc-2043, Lot#2612) for 1 hr. Then the cells were incubated at room temperature for one hour and transferred to 4°C overnight with one of the primary antibodies: monoclonal antibody to fibroblast surface protein (FSP-1, 1∶50 dilution, Cat# AM03212PU-N, Clone:1B10, Lot# T300, Acris, Germany) and anti-human thymic fibroblasts (TE7, 1∶50 dilution, Cat# sc-73603, Lot#12611). After the cells were washed the next day, they were incubated with secondary antibodies: goat-anti-mouse IgG-FITC (1∶100 dilution, Cat# sc-2010, Lot# D0312) and goat-anti-mouse IgG-TR (1∶50 dilution, Cat# sc-2781, Lot#E3012, Santa Cruz Biotech) for 2 hrs at room temperature, then washed three times with PBS. Immunostaining was analyzed with the confocal laser-scanning microscopy (NINDS, Confocal facility, NIH).

### iPSC Generation

We used a modification of the approach described in Sommer and Mostoslavsky to generate iPSCs from the postmortem fibroblasts [9]. The polycistronic plasmid pHAGE2-hSTEMCCA-loxp was used to produce lentiviral particles (LentiPhos HT, Clontech) that transduce OCT4, KLF4, C-MYC and SOX2. Fibroblasts were plated at 1.33×10^3^/cm^2^ in a T-75 flask the night before infection. For infection, fibroblast culture medium was removed and viral supernatant supplemented with 4 µg/ml polybrene was added to each T-75 of fibroblasts and removed 4 hrs later. The next day, the infection was repeated again. Virus-containing medium was removed again after 4 hrs and replaced with fresh culture medium. On day 5, cells were trypsinized and plated at a density of 868 cells/cm^2^ into 6-well plates containing irradiated mouse embryonic feeder cells (CF-1, GlobalStem). Infected human fibroblasts were allowed to attach overnight in fibroblast culture medium containing serum. The following morning, cells were fed with human pluripotent cell medium (DMEM:F12 (Invitrogen Cat# 11330-032) containing 20% Knockout Serum Replacement (KSR) (Invitrogen Cat# 10828-028), 1 mM glutamine (Invitrogen Cat# 25030-081), 0.1 mM β-mercaptoethanol (β-ME; Sigma), 1× non-essential amino acids (NEAA; Invitrogen Cat# 11140-050) and 4 ng/ml bFGF (R&D Systems)(Cat# 233-FB) and then fed every 48 hrs until day 10. After day 10, cells were fed everyday with MEF-conditioned medium to account for the progressive loss of feeders during the extended culture period. Potential colonies appeared between days 24 and 48 and were manually picked and expanded based on morphology. iPSC colonies were passaged every 5 to 6 days.

### Statistics

We used one-way ANOVA to analyze differences between scalp and dura fibroblast proliferation at 24 hrs and 48 hrs, using Statistica (Statsoft, Inc., version 8.0). We used logistic regression to identify differences in culture success, and to assess whether clinical covariates were associated with the odds success in the tissue-specific analyses. All reported odds ratios and 95% confidence intervals were calculated using these logistic regression models, and respective p-values for each odds ratio were calculated via Wald tests. We assessed potential confounders in Table 3 using chi-squared tests for categorical or binary covariates (using permutation to obtain p-values), and Wald statistics from linear regression for continuous covariates. We calculated matched odds ratios and employed McNemar’s Test on the paired samples from 53 individuals. The R statistical software (version 2.14.0) was used for these analyses.

## Supporting Information

Figure S1 **Expression of FSP-1 and TE7 proteins in both dura and scalp fibroblasts by immunofluorescence staining.** Upper Panel: Postmortem fibroblast morphology from dura and scalp samples from two different individuals under phase-contract microscopy. Lower panel: FSP-1 (green) and human thymic fibroblast (TE7) protein (red) were expressed in the cytoplasm of fibroblast cells in both dura and scalp samples from two different individuals. Original scale bars = 35 µm.
(TIF)

Figure S2 **Scatterplots of association between culture success in scalp and dura with PMI and BMI.** PMI =  postmortem interval; BMI = body mass index; outliers with BMI>60 were excluded.
(TIFF)
