# Supplementary figures and images for: Use of Postmortem Human Dura Mater and Scalp for Deriving Human Fibroblast Cultures
Source: PLoS One. 2012 Sep 27;7(9):e45282. doi: 10.1371/journal.pone.0045282 (PMC3459947; doi:10.1371/journal.pone.0045282)

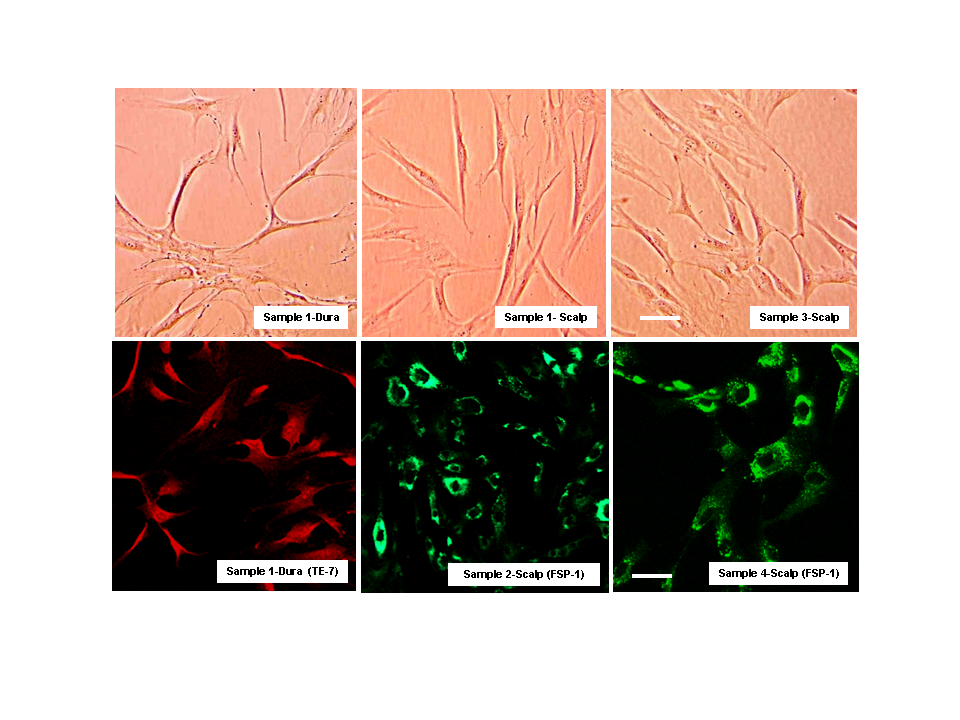

Supplement: Figure S1 — Expression of FSP-1 and TE7 proteins in both dura and scalp fibroblasts by immunofluorescence staining. Upper Panel: Postmortem fibroblast morphology from dura and scalp samples from two different individuals under phase-contract microscopy. Lower panel: FSP-1 (green) and human thymic fibroblast (TE7) protein (red) were expressed in the cytoplasm of fibroblast cells in both dura and scalp samples from two different individuals. Original scale bars = 35 µm. (TIF) [file pone.0045282.s001.tif]

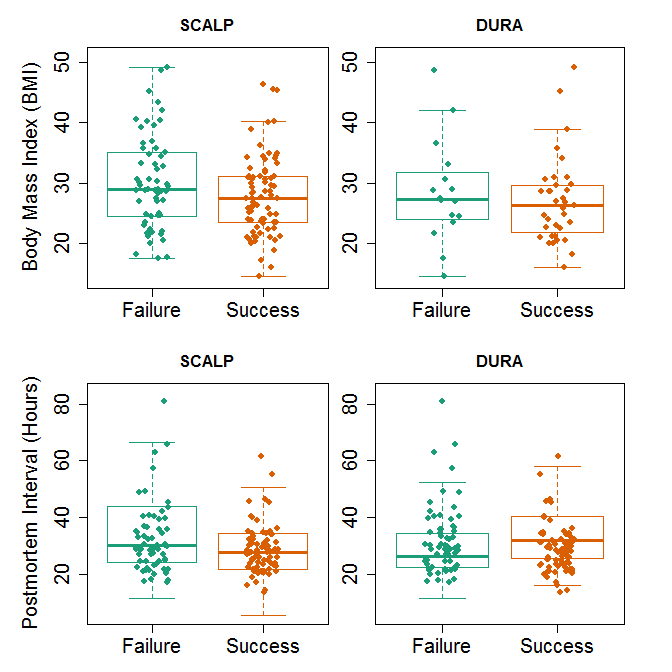

Supplement: Figure S2 — Scatterplots of association between culture success in scalp and dura with PMI and BMI. PMI = postmortem interval; BMI = body mass index; outliers with BMI>60 were excluded. (TIFF) [file pone.0045282.s002.tif]
